# Supplementary material for: Allosteric rescue of catalytically impaired ATP phosphoribosyltransferase variants links protein dynamics to active-site electrostatic preorganisation
Source: Nat Commun. 2022 Dec 9;13:7607. doi: 10.1038/s41467-022-34960-9 (PMC9734150; doi:10.1038/s41467-022-34960-9)
Supplement: Supplementary file 1 — Supplementary Information [file 41467_2022_34960_MOESM1_ESM.pdf]

Supplementary information

Allosteric rescue of catalytically impaired ATP phosphoribosyltransferase variants links protein dynamics to active-site electrostatic preorganisation

Gemma Fisher[1], Marina Corbella[2], Magnus S. Alphey[1], John Nicholson[1], Benjamin J. Read[1], Shina C. L. Kamerlin[2,3,*], and Rafael G. da Silva[1,*]

[1]School of Biology, Biomedical Sciences Research Complex, University of St Andrews, St Andrews, KY16 9ST, UK.

[2]Science for Life Laboratory, Department of Chemistry – BMC, Uppsala University, S-751 23 Uppsala, Sweden.

[3]School of Chemistry and Biochemistry, Georgia Institute of Technology, 901 Atlantic Drive NW, Atlanta, GA 30332, USA

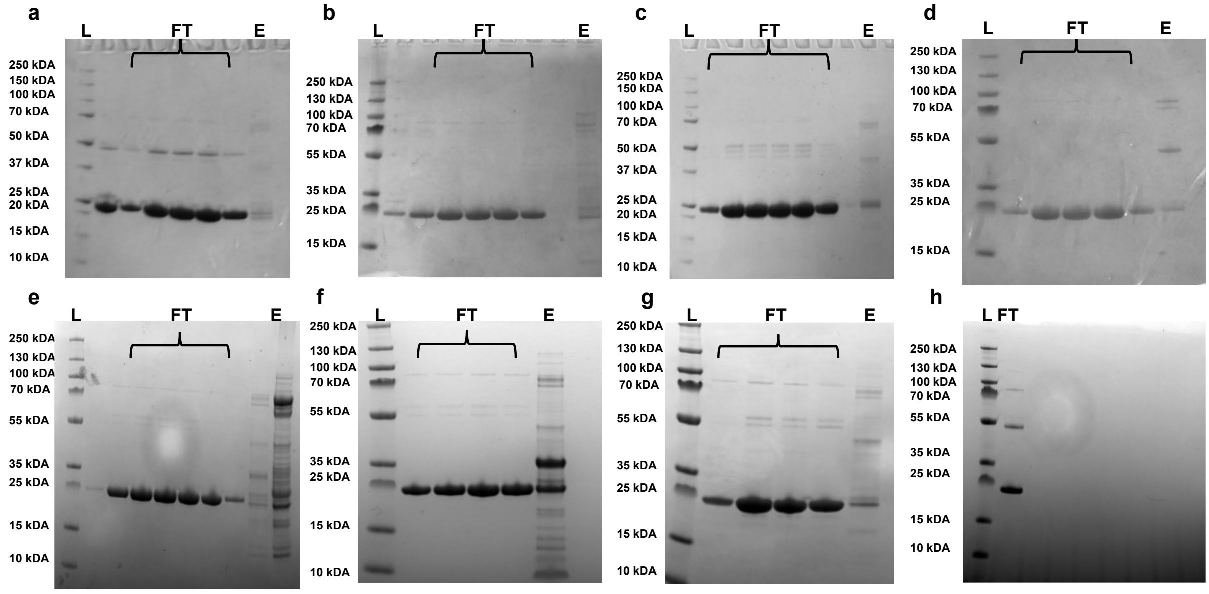

Supplementary Figure 1 SDS-PAGE analysis of purified *Pa*HisG$_S$ mutants eluted from the HisTrap FF column in the second chromatography. **a** C115A. **b** C115S. **c** D179A. **d** D179N. **e** R32A. **f** R56A. **g** R56A/K57A. **h** R32A/R56A/K57A. Lanes are as follows: L is the MW marker; FT is the flowthrough, which was pooled; E is the elution, which was discarded. The MW marker is either the PageRuler Plus Prestained (**b, d**, **e**, **f**, **g**, **h**) or the Precision Plus All Blue Protein Prestained (**a**, **c**). Images **a** – **d** and **h** are representative of one batch of the respective *Pa*HisG$_S$ variant preparation; images **e** – **g** are representative of three independent batches of the respective *Pa*HisG$_S$ variant preparation.

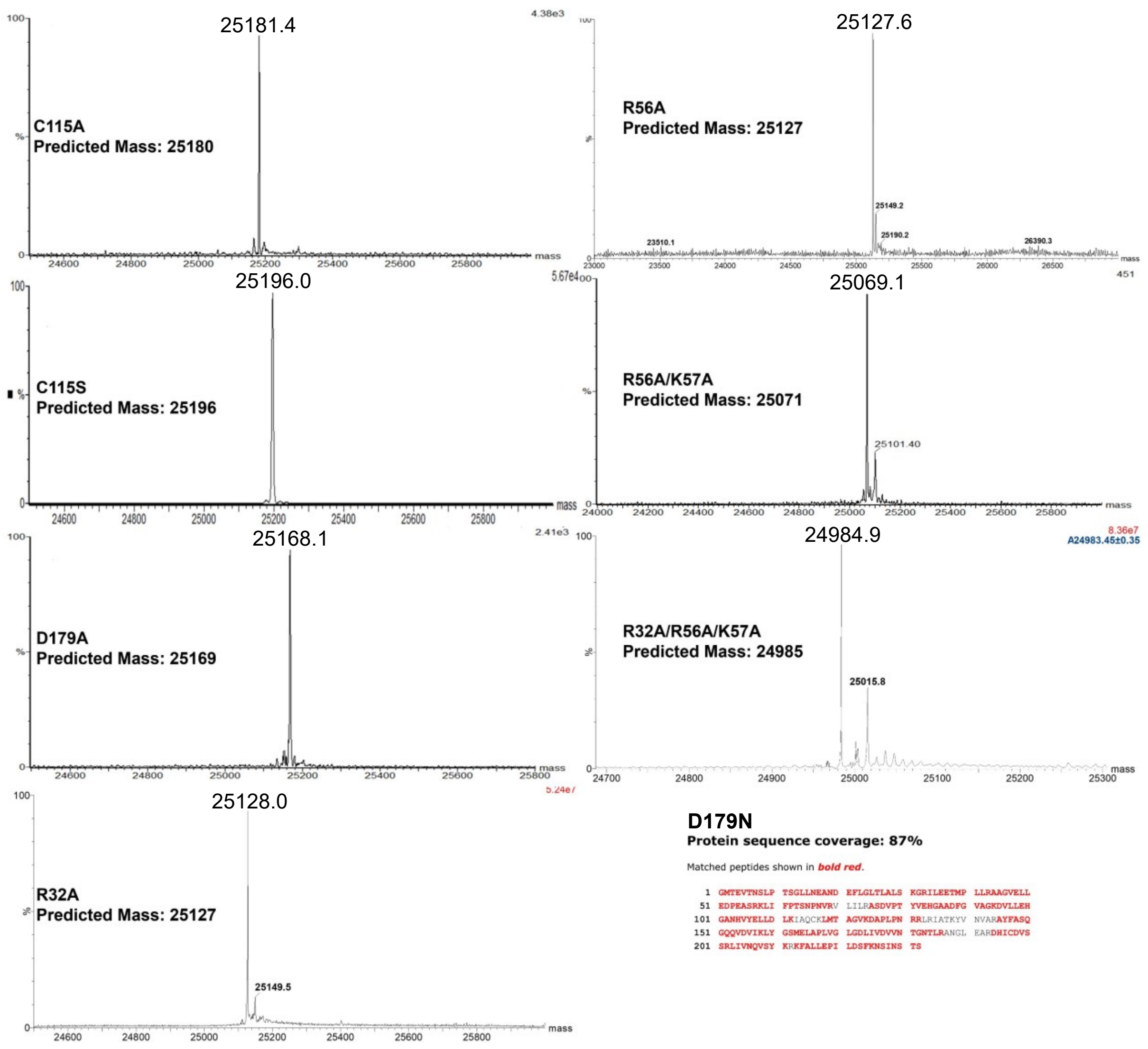

Supplementary Figure 2 ESI/TFO-MS analysis of *Pa*HisG$_S$ mutants. For the C115A, C115S, D179A, R32A, R56A, R56A/K57A, and R32A/R56A/R32A mutants, the intact molecular mass was determined. For the D179N mutant, peptide mapping of the trypsin-digested protein was carried out.

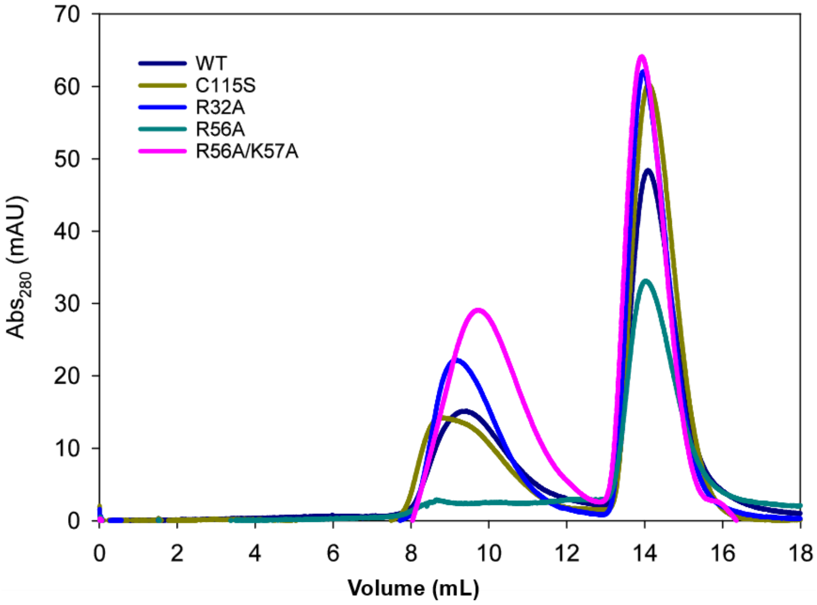

Supplementary Figure 3 Analytical size-exclusion chromatography elution profile of *Pa*HisG$_S$ mutants. The peaks centred at ~14 mL indicate a dimer, while the peaks at ~9 mL reflect a higher oligomeric state. Source data are provided as a Source Data file.

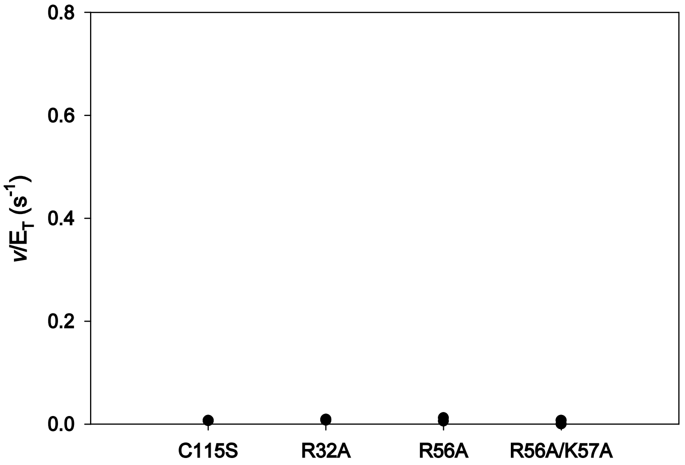

Supplementary Figure 4 Apparent rate constants for reactions catalysed by *Pa*HisG$_S$ mutants in the presence of 20 μM BSA. All data points are shown for duplicate measurements. The scale in the *y*-axis is the same as that of Figure 2c. Source data are provided as a Source Data file.

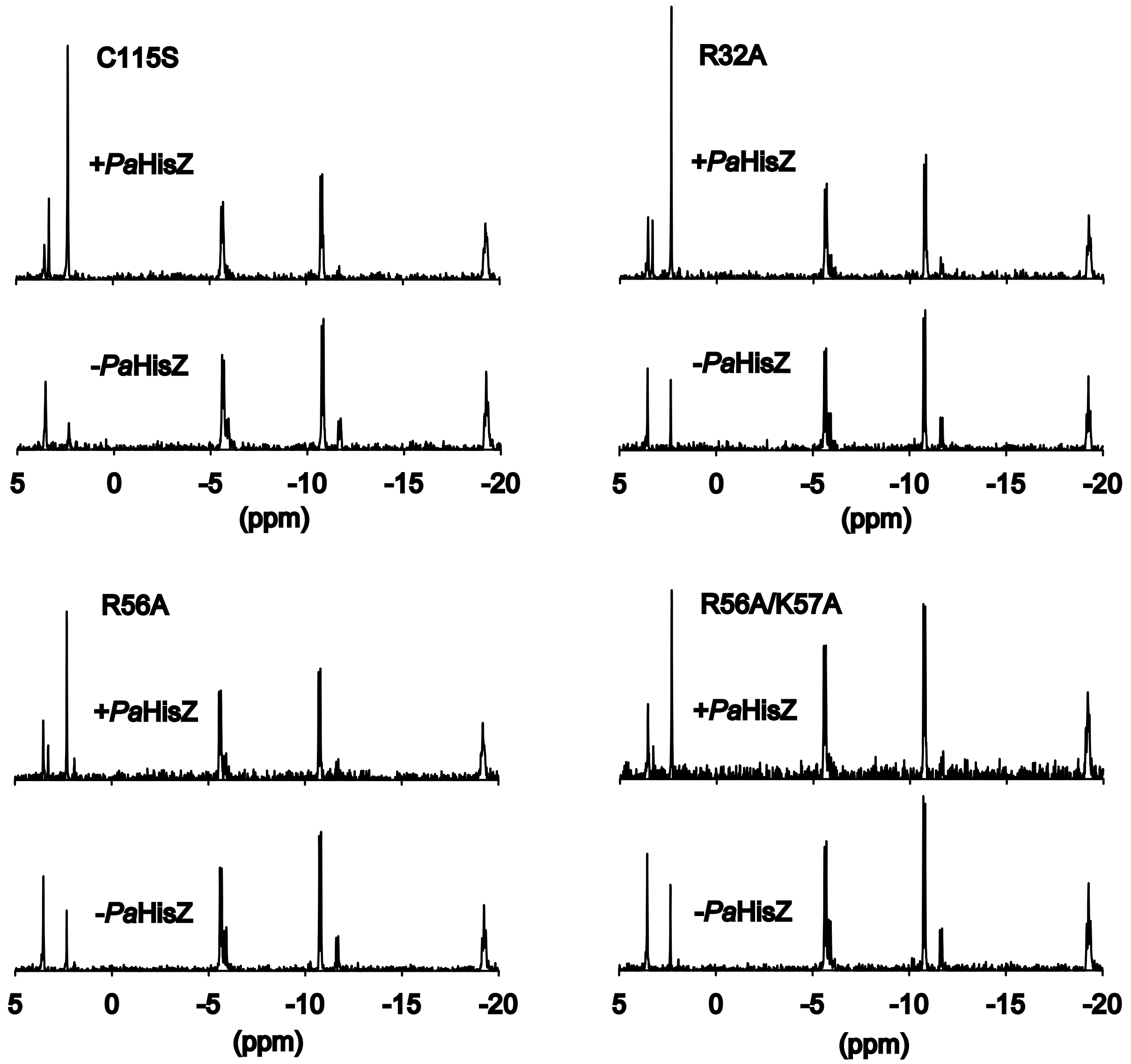

Supplementary Figure 5 Analysis by ^{31}P-NMR spectroscopy of the reaction catalysed by *Pa*HisG$_S$ mutans in the presence and absence of *Pa*HisZ. The chemical shift at ~2.3 ppm corresponds to inorganic phosphate, which is produced by hydrolysis of the PP$_i$ co-product by a pyrophosphatase used in the assay to drive forward the highly unfavourable ATPPRT reaction equilibrium.

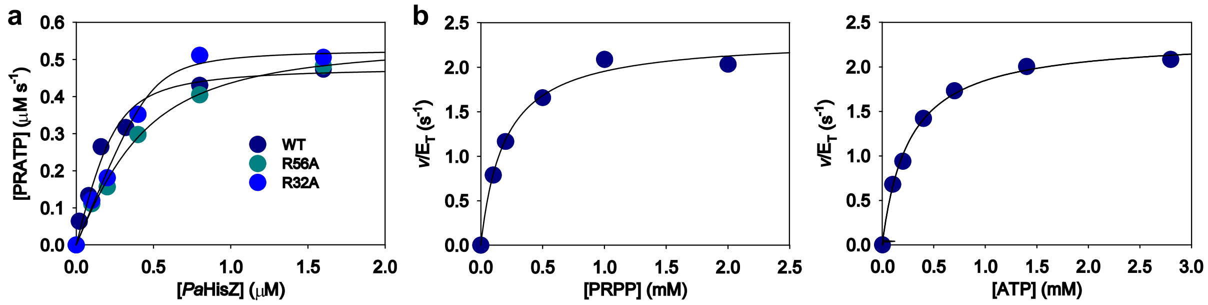

Supplementary Figure 6 Binding and kinetics of His-tagged *Pa*HisZ **a** Dependence of rate of reaction catalysed by *Pa*HisG$_S$ variants on the concentration of His-tagged *Pa*HisZ. Data are the mean of two independent measurements. Best fit of the data to equation (3) is shown as solid line. The apparent K_D is 0.06 ± 0.03 μM for WT-*Pa*ATPPRT, 0.03 ± 0.02 μM for R32A-*Pa*ATPPRT, and 0.2 ± 0.1 μM for R56A-*Pa*ATPPRT. Source data are provided as a Source Data file. **b** Substrate saturation curves for WT-*Pa*ATPPRT with His-tagged *Pa*HisZ. Data are the mean of two independent measurements. Lines are best fit of the data to equation (1). Apparent steady-state kinetic apparent k_{cat}, K_{PRPP} and K_{ATP} are 2.33 ± 0.05 s^{-1}, 0.19 ± 0.03 mM, and 0.26 ± 0.02 mM, respectively. Source data are provided as a Source Data file.

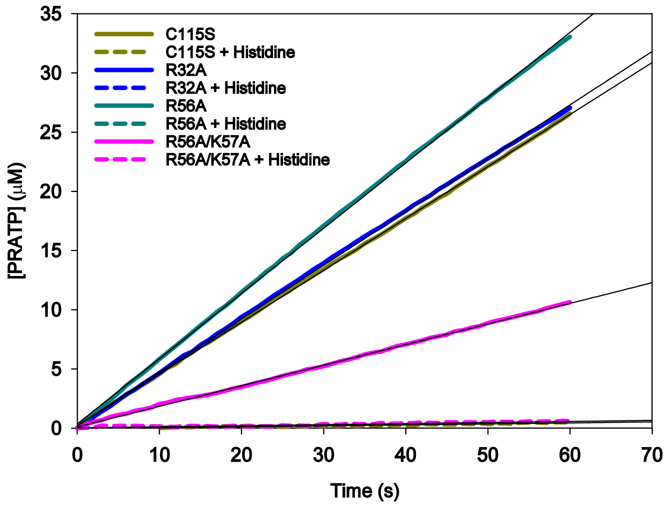

Supplementary Figure 7 Product formation time course of the reaction catalysed by the rescued *Pa*ATPPR mutants in the presence and absence of 1 mM histidine. Traces are averages of two independent measurements, and black lines are linear regressions of the data. Source data are provided as a Source Data file.

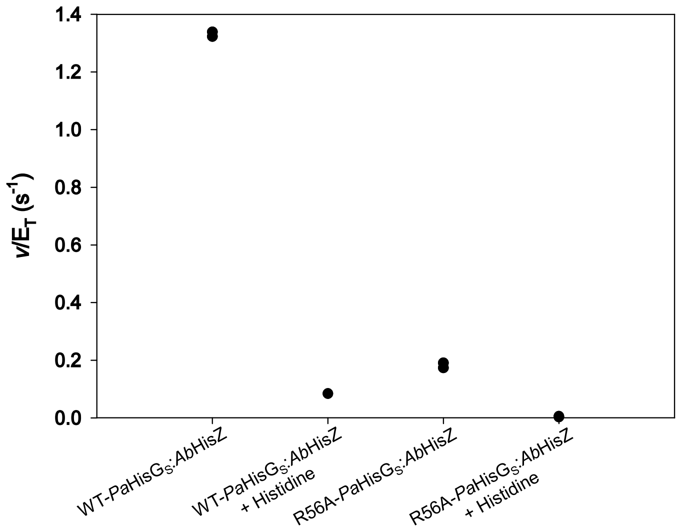

Supplementary Figure 8 Apparent rate constants for *Pa*HisG$_S$:*Ab*HisZ-catalysed reaction in the presence and absence of 1 mM histidine. All data points are shown for duplicate measurements. Source data are provided as a Source Data file.

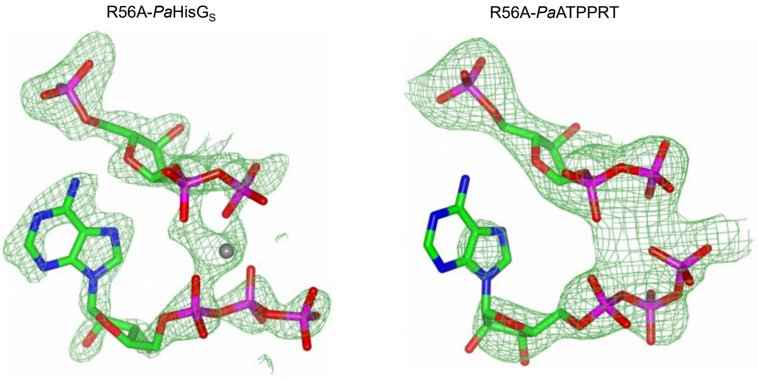

Supplementary Figure 9 $F_{obs} - F_{calc}$ electron density omit maps at 2.5 σ for the substrates ATP and PRPP in the structures of R56A-*Pa*HisG$_S$ and R56A-*Pa*ATPPRT. Ligands are depicted as sticks with oxygen in red, nitrogen in blue, phosphorus in magenta, and carbon in green. The magnesium atom is depicted as a sphere.

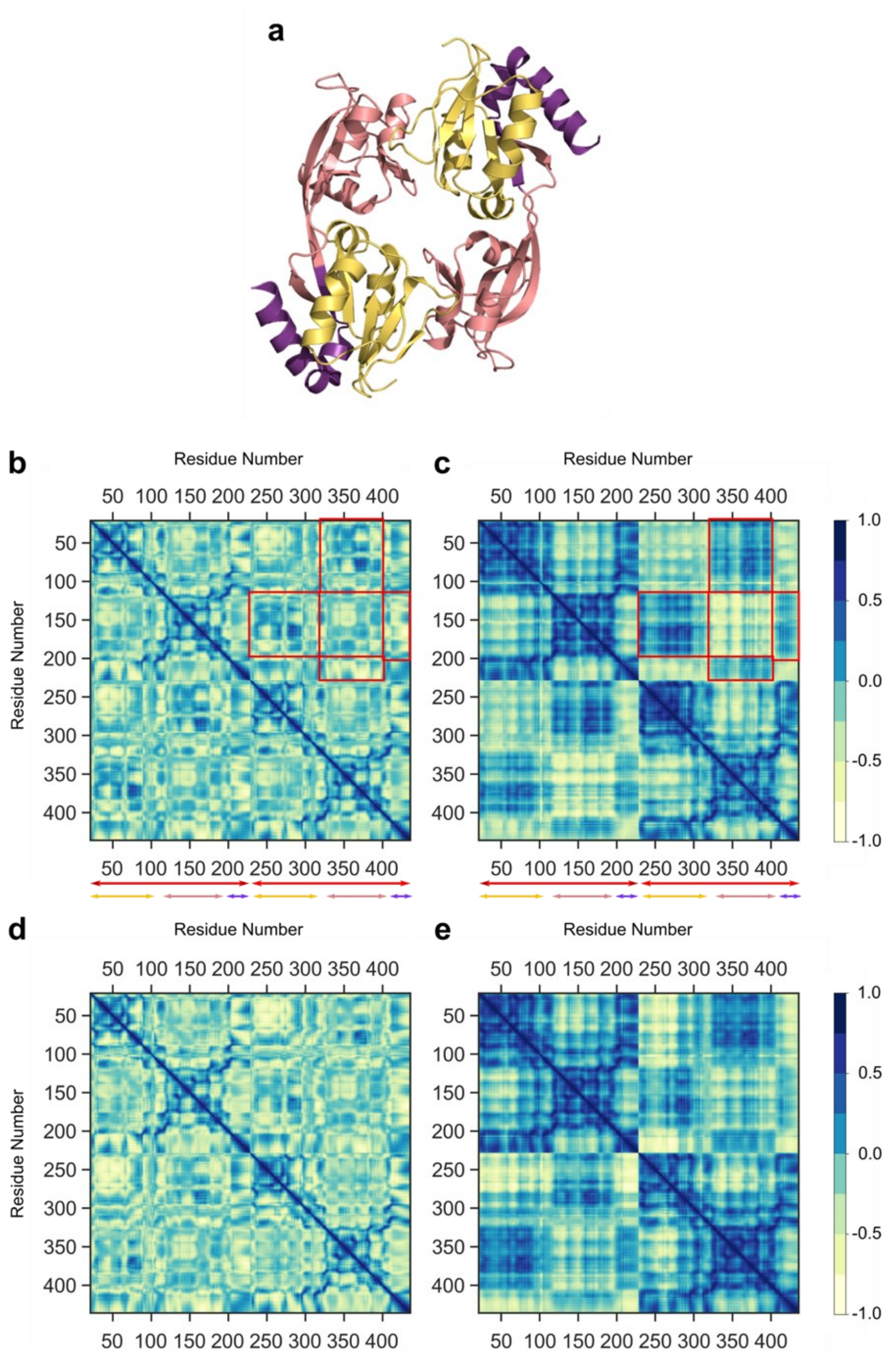

Supplementary Figure 10 Dynamic cross-correlation matrices of the Cα-atoms. **a** color-coded ribbon diagram of the *Pa*HisG$_S$ dimer. **b** Nonactivated WT-*Pa*HisG$_S$ dimer. **c** Activated WT-*Pa*HisG$_S$ dimer. **d** Nonactivated R56A-*Pa*HisG$_S$ dimer. **e** Activated R56A-*Pa*HisG$_S$ dimer. The DCCM plots were generated from the corresponding correlation matrices, based on 10 × 500 ns (nonactivated enzyme) and 5 × 500 ns (activated enzyme) of simulation time per system.

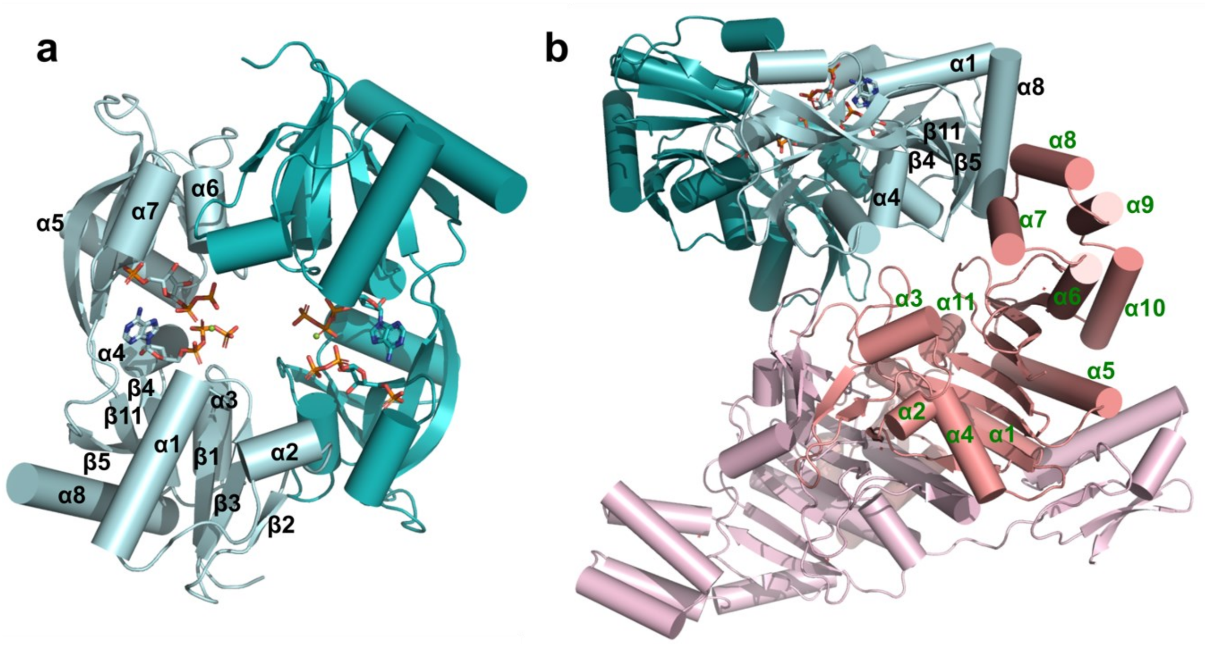

Supplementary Figure 11 Secondary structure numbering of *Pa*ATPPRT (PDB ID: 6FU2). **a** Cartoon representation of the *Pa*HisG$_S$ dimer. **b** Cartoon representation of the *Pa*HisG$_S$ dimer in complex with one subunit of *Pa*HisZ tetramer. Substrates are depicted as sticks, and Mg^{2+} as spheres.

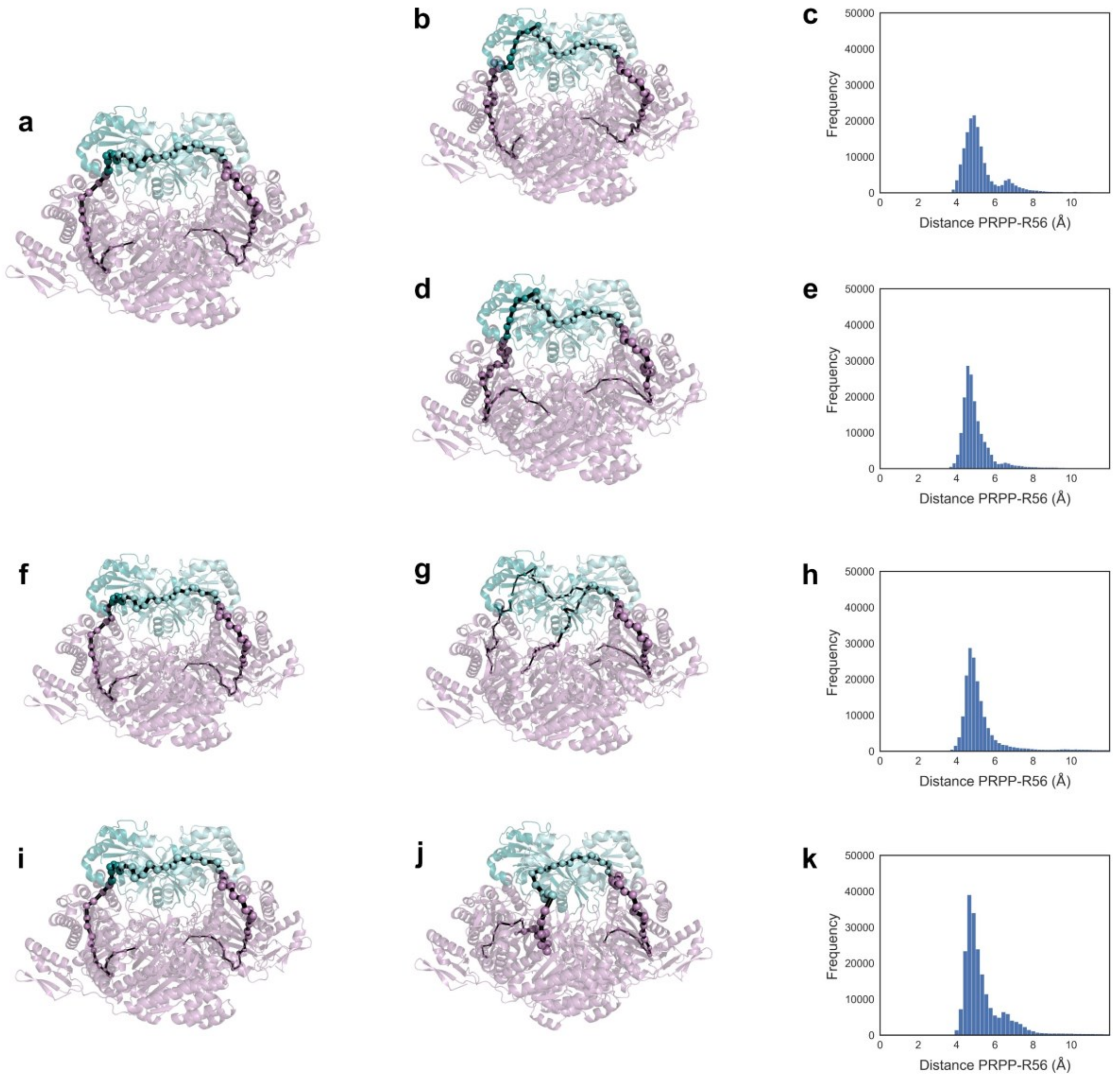

Supplementary Figure 12 Node-weakening analysis of the *Pa*ATPPRT SPM. **(a)** SPM upon removal of Y105 from *Pa*HisG$_S$. **(f)** SPM upon removal of N185 from *Pa*HisZ. **(i)** SPM upon removal of K186 from *Pa*HisZ. **(b, d, g** and **j) (b)** SPM of Y105A-*Pa*HisG$_S$. **(d)** SPM of Y105F-*Pa*HisG$_S$. **(g)** SPM of N185A-*Pa*HisZ. **(j)** SPM of K186D-*Pa*HisZ. **(c, e, h** and **k)** Distribution of PRPP – R56 distances in **(c)** Y105A-*Pa*ATPPRT, **(e)** Y105F-*Pa*ATPPRT, **(h)** N185A-*Pa*ATPPRT, and **(k)** K186D-*Pa*ATPPRT.

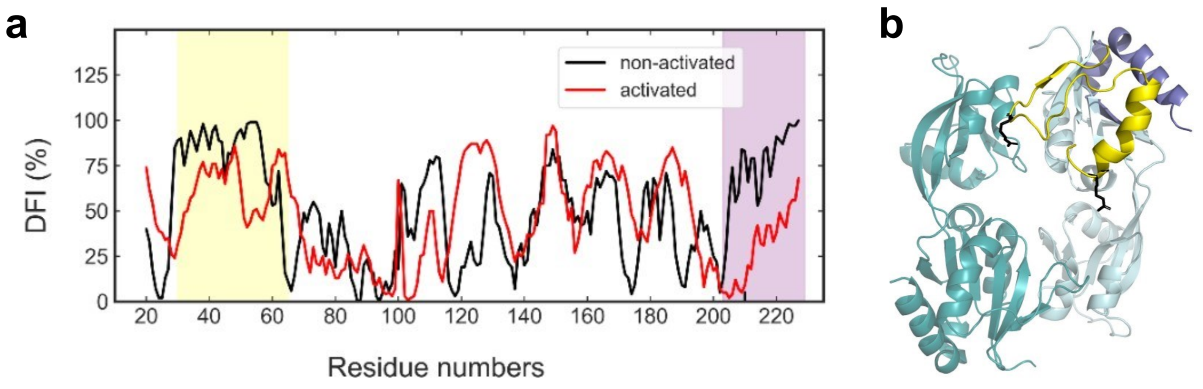

Supplementary Figure 13 Dynamical flexibility indices. a DFI of monomeric subunits of nonactivated and activated *Pa*HisG$_S$, calculated based on 10 × 500 ns of MD simulations of each system. The higher the DFI score, the more flexible the protein region. **b** Representative structure of *Pa*HisG$_S$ onto which the two regions highlighted in yellow and purple in the DFI plot are mapped. The region highlighted in yellow encompass R32 and R56, whose side chains are depicted in stick models in black. The region highlighted in purple includes the interface with *Pa*HisZ.

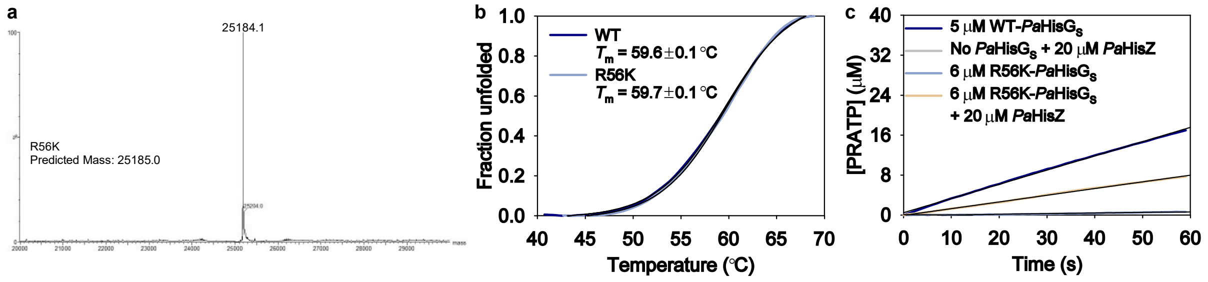

Supplementary Figure 14 Biochemical characterisation of R56K-*Pa*HisG$_S$. a ESI/TFO-MS analysis of R56K-*Pa*HisG$_S$. **b** DSF-based thermal denaturation of WT- and R56K-*Pa*HisG$_S$. Traces are averages of three independent measurements. Lines of best fit to equation (2) are in black. Source data are provided as a Source Data file. **c** PRATP formation time course catalysed by WT- and R56K-*Pa*HisG$_S$. Traces are averages of two measurements. Black lines are linear regressions of the data. Source data are provided as a Source Data file.

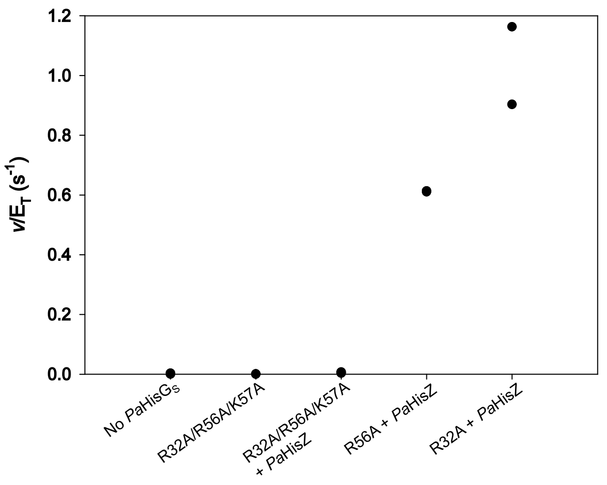

Supplementary Figure 15 Apparent rate constants for single and triple mutants of *Pa*HisG$_S$. All data points are shown. Source data are provided as a Source Data file.

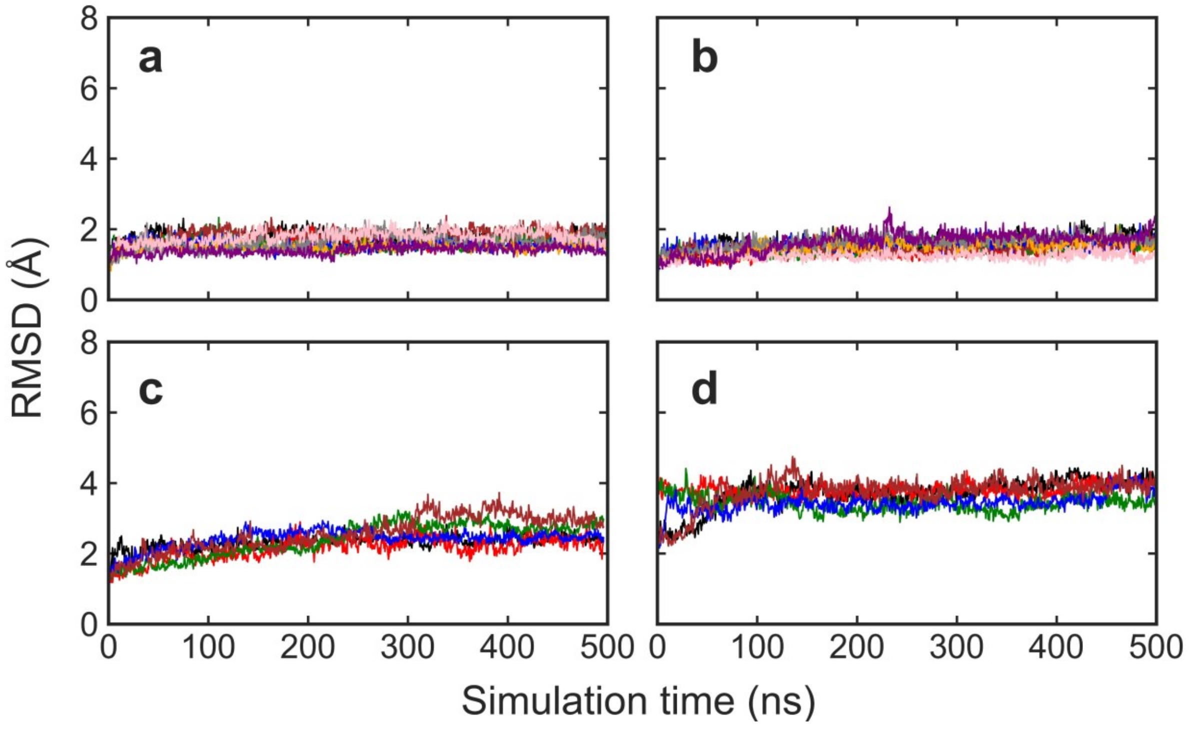

Supplementary Figure 16 RMSD of all backbone heavy atoms from MD simulations. **a** WT-*Pa*HisG$_S$. **b** R56A-*Pa*HisG$_S$. **c** WT-*Pa*ATPPRT. **d** R56A-*Pa*ATPPRT. All individual simulations are shown.

Supplementary Table 1 Apparent rate constants (mean ± fitting error) for *Pa*HisG$_S$ variants from reactions monitored for 56 s.

***Pa*HisG$_S$**	**v/E$_T$ (s^{-1})**
**WT**	0.0586 ± 0.0002
**C115A**	0.0134 ± 0.0002
**C115S**	N. d.*
**D179A**	0.0256 ± 0.0001
**D179N**	0.0164 ± 0.0002
**R32A**	N. d.*
**R56A**	N. d.*
**R56A/K57A**	N. d.*

*No product formation detected above background noise.

Supplementary Table 2 Apparent steady-state kinetic parameters (mean ± fitting error) for WT-, C115A-, D179A-, and D179N-*Pa*HisG$_S$.

***Pa*HisG$_S$**	**k_{cat} (s^{-1})**	**K_{PRPP} (mM)**	**K_{ATP} (mM)**
**WT**	0.081 ± 0.003	0.40 ± 0.06	1.2 ± 0.2
**C115A**	0.020 ± 0.001	0.42 ± 0.07	2.2 ± 0.3
**D179A**	0.030 ± 0.001	0.16 ± 0.01	0.8 ± 0.2
**D179N**	0.020 ± 0.002	0.13 ± 0.02	1.7 ± 0.5

Supplementary Table 3 DSF-based T_m (mean ± fitting error) for *Pa*HisG$_S$ mutants in the presence and absence of PRPP.

*Pa*HisG$_S$	(−PRPP) T_m (°C)	(+PRPP) T_m (°C)	ΔT_m (°C)
**WT**	57.83 ± 0.07	65.4 ± 0.09	+7.8 ± 0.2
**C115S**	56.75 ± 0.09	63.53 ± 0.09	+8.8 ± 0.2
**R32A**	60.39 ± 0.06	63.81 ± 0.06	+3.4 ± 0.1
**R56A**	58.11 ± 0.07	62.59 ± 0.07	+4.5 ± 0.2
**R56A/K57A**	56.77 ± 0.07	60.3 ± 0.1	+3.5 ± 0.2

Supplementary Table 4 X-Ray diffraction data collection and refinement statistics.

	**R56A-*Pa*HisG$_S$**	**R56A-*Pa*ATPPRT**
**Data Collection**		
**PDB ID**	7Z8U	7Z6R
**Space group**	I2	C2
**Cell dimensions**		
a,b,c **(Å)**	70.09, 33.90, 89.27	102.16, 145.53, 93.81
**α,β,γ (°)**	90.00, 103.52, 90.00	90.00, 102.48, 90.00
**Resolution (Å)**	22.01 – 2.00 (2.05 – 2.00)	56.97 – 2.55 (2.61 – 2.55)
**R_{merge}**	0.11 (0.36)	0.17 (3.17)
**I/σI**	7.2 (2.5)	10.9 (0.8)
**Completeness (%)**	93.8 (88.2)	99.9 (99.3)
**Redundancy**	3.0 (2.6)	6.5 (7.1)
**CC ½**	0.99 (0.84)	0.99 (0.28)
**Refinement**		
**Resolution (Å)**	22.01 – 2.00	56.97 - 2.55
**No. reflections**	12411	41555
**R_{work}/R_{free} (%)**	22.1/26.8	23.3/27.6
**No. atoms**		
**Protein**	1573	8431
**Ligand/ion**	54	106
**Water**	42	16
**B-factors**		

**Protein**	21.04	79.9
**Ligand/ion**	40.01	104.8
**Water**	20.24	58.3
**r.m.s. deviations**		
**Bond lengths (Å)**	0.008	0.005
**Bond angles (°)**	1.623	1.374
**Ramachandran**		
**Favoured (%)**	97	96
**Allowed (%)**	3	4
**Outliers (%)**	0	0

Numbers in brackets are from the highest resolution shell.

Supplementary Table 5 Pair-wise residue details for the calculated SPM from simulations of nonactivated *Pa*HisG$_S$, and the corresponding weight of each edge.[a]

Residue 1		Residue 2		Weight Path
VAL	90	ALA	91	3.9792
GLY	92	LYS	93	3.1818
LYS	93	LEU	96	3.3612
LEU	96	GLU	98	3.1168
VAL	204	ILE	203	3.8564
ILE	203	LEU	202	3.8508
LEU	202	ARG	201	3.7237
ARG	201	SER	200	3.6055
SER	200	SER	199	4.0759
SER	199	VAL	198	3.1039
LYS	116	LEU	117	3.2349
LEU	117	MET	118	3.0098
MET	118	THR	119	3.1900
LEU	173	ASP	172	4.1731
ASP	172	GLY	171	4.0045
GLY	171	LEU	170	7.6669
LEU	170	VAL	168	7.6678
VAL	168	ALA	85	8.6954
ALA	85	ALA	86	6.4069
ALA	86	ASP	87	4.4410
ASN	86	VAL	87	4.4616

ASN	86	ALA	85	6.8237
ALA	85	VAL	168	10.0000
VAL	168	LEU	170	8.9771
LEU	170	GLY	171	8.9771
GLY	171	ASP	172	6.3200
ASP	172	LEU	173	4.8177
LEU	173	ALA	120	3.2546
ALA	120	THR	119	3.5366
THR	119	MET	118	3.8555
MET	118	LEU	117	3.7686
LEU	117	LYS	116	3.8555
LYS	116	VAL	198	3.1150
VAL	198	SER	199	3.7203
SER	199	SER	200	5.2555
SER	200	ARG	201	5.4306
ARG	201	LEU	202	4.8720
LEU	202	ILE	203	4.5818
ILE	203	VAL	204	4.6289
GLY	171	ALA	135	3.0196
ALA	135	THR	136	3.2310
THR	136	TYR	138	3.3115
TYR	138	GLU	98	3.3714
GLU	98	LEU	96	3.9715
LEU	96	LYS	93	4.2326
LYS	93	GLY	92	3.5709
GLY	92	ALA	91	3.7023
ALA	91	VAL	90	3.7036

[a]Different colors correspond to different chains in PDB ID: 6FCT.

Supplementary Table 6 Pair-wise residue details for the calculated SPM from simulations of activated *Pa*HisGs, and the corresponding weight of each edge.[a]

Residue 1		Residue 2		Weight Path
ARG	68	THR	62	4.8485
VAL	69	ARG	68	3.6072
LEU	26	VAL	69	4.4229
ALA	86	LEU	26	3.4353

LEU	26	GLY	89	4.6976
GLY	89	VAL	90	6.6853
VAL	90	ALA	91	3.7399
ALA	91	GLY	92	3.7474
GLY	92	VAL	95	3.4459
VAL	95	GLU	98	3.4487
VAL	90	ARG	201	4.1312
ARG	201	SER	200	7.5503
SER	200	SER	199	7.6430
SER	199	VAL	198	7.5386
VAL	198	LYS	116	7.0621
LYS	116	LEU	117	6.9939
LEU	117	MET	118	6.9511
MET	118	THR	119	7.1750
THR	119	ALA	120	7.1458
GLU	98	TYR	138	3.2742
TYR	138	THR	136	3.3711
ALA	120	LEU	173	6.3377
LEU	173	ASP	172	9.3500
ASP	172	PRO	166	10.0000
PRO	166	VAL	168	8.7992
VAL	168	ALA	85	9.1712
ALA	85	ALA	86	8.4741
ALA	86	THR	25	3.6802
THR	25	ARG	68	3.0127
ARG	68	THR	62	4.5654
ALA	86	ASP	87	5.0212
ASP	87	PHE	88	3.5564
PHE	88	GLY	89	3.0931
ALA	86	ALA	85	6.6750
ALA	85	VAL	168	7.1952
VAL	168	PRO	166	3.5400
PRO	166	ASP	172	3.9487
ASP	172	LEU	173	4.2582
ALA	120	THR	119	3.9304
THR	119	MET	118	5.0969
MET	118	LEU	117	5.6872
LEU	117	LYS	116	5.6914
LYS	116	VAL	198	5.4384
VAL	198	LYS	93	4.9012
LYS	93	GLY	92	6.0395
GLY	92	ALA	91	6.8039

ALA	91	VAL	90	6.8161
ILE	203	LEU	202	3.7587
LEU	202	ARG	201	3.6689
VAL	90	GLY	89	4.8490

[a]Different colors correspond to different chains in PDB ID: 6FU2.

Supplementary Table 7 Pair-wise residue details for the calculated SPM from simulations of *Pa*ATPPRT, and the corresponding weight of each edge.[a]

Residue 1		Residue 2		Weight Path
ARG	95	TYR	33	3.0443
TYR	33	LEU	29	3.1903
KEU	29	GLN	26	4.2941
GLN	26	GLN	23	4.3405
GLN	23	LEU	20	4.3876
LEU	20	GLN	17	4.4148
GLN	17	ALA	14	4.4461
ALA	14	LEU	35	4.4438
LEU	35	TYR	96	5.9244
TYR	96	GLU	122	5.9200
GLU	122	THR	305	6.6620
THR	305	ARG	287	8.0728
ARG	287	ILE	269	8.6435
ILE	269	HIS	158	8.5982
HIS	158	VAL	257	8.5518
VAL	257	THR	258	8.6687
THR	258	TYR	183	8.9347
TYR	183	ASN	185	8.9093
ASN	185	TYR	105	10.0000
TYR	105	VAL	204	8.9797
VAL	204	PHE	88	8.8591
PHE	88	ASP	87	8.4124
ASP	87	ALA	86	8.5805
ALA	86	ALA	85	9.0319
ALA	85	VAL	168	9.1151
VAL	168	PRO	166	9.0476
PRO	166	ASP	172	9.2263

ASP	172	LEU	173	9.1944
LEU	173	ALA	120	8.7130
ALA	120	THR	119	8.8404
THR	119	MET	118	8.8578
MET	118	LEU	117	8.8274
LEU	117	LYS	116	8.8368
LYS	116	VAL	198	8.8550
VAL	198	SER	199	8.9164
SER	199	SER	200	8.9271
SER	200	ARG	201	8.8998
ARG	201	LEU	108	8.2753
LEU	108	LEU	107	8.8351
LEU	107	LEU	188	9.9424
LEU	188	LEU	191	9.6987
LEU	191	VAL	194	9.6760
VAL	194	CYS	195	9.6452
CYS	195	LEU	198	9.3265
LEU	198	PRO	199	9.3380
PRO	199	ALA	169	9.3192
ALA	169	LEU	168	9.3083
LEU	168	LEU	165	9.3027
LEU	165	ARG	164	9.2840
ARG	164	THR	160	8.9520
THR	160	GLY	268	8.2212
GLY	268	THR	287	8.2208
THR	287	THR	305	8.3787
THR	305	GLU	122	5.5945
GLU	122	TYR	96	5.5705
TYR	96	LEU	35	5.5648
LEU	35	PHE	11	4.4578
PHE	11	ASP	13	4.4617
ASP	13	LYS	16	4.4530
LYS	16	LEU	20	4.5380
LEU	20	GLN	23	4.5299
GLN	23	GLN	26	4.5000
GLN	26	ILE	29	4.4702
ILE	29	TYR	33	3.3131
TYR	33	ARG	95	3.0655
ARG	95	ILE	123	3.0061

[a]Different colors correspond to different chains in PDB ID: 6FU2.

Supplementary Table 8 Difference in the CPL (characteristic path length) upon removing all contacts from a given interface residue.

*Pa*HisGs		
**Residue removed**[a]	**CPL (Å)**	**ΔCPL**[b] **(Å)**
Tyr 105	26.899	0.847
Glu 106	26.060	0.008
Leu 107	26.080	0.028
Leu 108	26.082	0.030
Asp 109	26.058	0.006
Tyr 105	26.215	0.163
Glu 106	26.056	0.004
Leu 107	26.068	0.016
Leu 108	26.057	0.005
Asp 109	26.056	0.004
***Pa*HisZ**		
**Residue removed**[a]	**CPL (Å)**	**ΔCPL**[b] **(Å)**
Ala 184	26.055	0.003
Asn 185	26.209	0.157
Lys 186	26.055	0.003
Asn 187	26.107	0.055
Leu 188	26.098	0.046
Pro 189	26.054	0.002
Glu 190	26.054	0.002
Ala 184	26.059	0.007
Asn 185	26.059	0.007
Lys 186	26.889	0.837
Asn 187	26.060	0.008
Leu 188	26.114	0.062
Pro 189	26.057	0.005
Glu 190	26.057	0.005

[a]Different residue colors correspond to different chains in PDB ID: 6FU2.

[b]The CPL for WT-*Pa*ATPPRT is 26.052 Å.

Supplementary Table 9 Primers used for site-directed mutagenesis of *Pa*HisG$_S$.

***Pa*HisGs Mutation**	**Forward Primer**	**Reverse Primer**
**C115A**	5′ - TTGCGCAGGCTAAACTGATGACCGCCGGTGTCAAAGACG - 3′	5′ - ATCAGTTTAGCCTGCGCAATCTTCAGATCCAACAGTTCATAAACGTGGTTC - 3′
**C115S**	5′ - TTGCGCAGTCTAAACTGATGACCGCCGGTGTCAAAGACG - 3′	5′ - ATCAGTTTAGACTGCGCAATCTTCAGATCCAACAGTTCATAAACGTGGTTCG - 3′
**D179A**	5′ - TCGTGGCCACCGGTAATACGCTGCGTG - 3′	5′ - ACCGGTGGCCACGACGTCAACAATCAGGTCACCCAGGC -3′
**D179N**	5′ - GTCGTGAACACCGGTAATACGCTGCGTGCGAACGG - 3′	5′ - CCGGTGTTCACGACGTCAACAATCAGGTCACCCAGGC -3′
**R32A**	5′ - AAGGGTGCCATCCTGGAAGAGACTATGCCGCTGTTGCG-3′	5′ - CAGGATGGCACCCTTGCTCAGTGCCAGGGTCAGACC-3′
**R56A**	5′-GCGCTAAGCTGATCTTCCCGACCAGCAACCCTAATGTG-3′	5′-GGAAGATCAGCTTAGCGCTCGCTTCCGGATCTTCCAGC-3′.
**R56A/K57A**	5′-GCGCTGCGCTGATCTTCCCGACCAGCAACCCTAATGTG-3′	5′-GAAGATCAGCGCAGCGCTCGCTTCCGGATCTTCCAGC-3′
**R56K**	5′-GCAAAAAGCTGATCTTCCCGACCAGCAACCCTAATGTG-3′	5′-GGAAGATCAGCTTTTTGCTCGCTTCCGGATCTTCCAG-3′

Supplementary Table 10 Non-standard force field parameters used to describe the substrate PRPP in the conventional MD simulations.

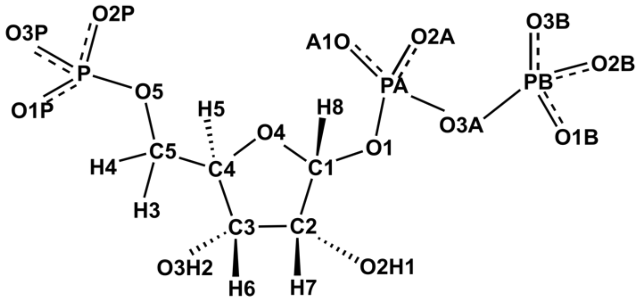

Atom Name	Atom type	Charge	Atom Name	Atom type	Charge
**H1**	HO	0.434144	O3	OH	-0.723957
**H2**	HO	0.471932	O4	OS	-0.605498
**H3**	H1	-0.011850	O5	OS	-0.445750
**H4**	H1	-0.011850	P	P	1.288976
**H5**	H1	0.094980	O1P	O3	-0.983784
**H6**	H1	-0.004183	O2P	O3	-0.983784
**H7**	H1	-0.028089	O3P	O3	-0.983784
**H8**	H2	0.010573	PA	P	1.195239
**C1**	CT	0.496695	O1A	O2	-0.872464
**C2**	CT	0.074132	O2A	O2	-0.872464
**C3**	CT	0.095379	O3A	OS	-0.540575
**C4**	CT	0.399838	PB	P	1.234914
**C5**	CT	0.111590	O1B	O3	-0.970435
**O1**	OS	-0.292571	O2B	O3	-0.970435
**O2**	OH	-0.636483	O3B	O3	-0.970435

Supplementary Table 11 Non-standard force field parameters used to describe the substrates in the conventional MD simulations.

**Restraint Index**	**Atom ID**	**Distances (Å)**				**Restraints (kcal mol^{-1} Å^{-2})**	
		***r*^{1}**	***r*^{2}**	***r*^{3}**	***r*^{4}**	***rk*^{2}**	***rk*^{3}**
**Distance 1**	PRP@P Asp179@N	0.0	3.0	4.2	4.5	0.0	10.0
**Distance 2**	PRP@P Thr180@N	0.0	3.0	4.2	4.5	0.0	10.0
**Distance 3**	PRP@P Thr183@N	0.0	3.0	4.2	4.5	0.0	10.0
**Distance 4**	PRP@O3 Asp176@OD2	0.0	2.5	3.0	3.5	0.0	10.0
**Distance 5**	ATP@O3* Asp94@CG	0.0	3.0	3.7	4.5	0.0	10.0
